# Role of osteoporosis and diabetes on facial and long bone fracture healing under various loading conditions

**DOI:** 10.6026/973206300213305

**Published:** 2025-09-30

**Authors:** Pradeep V. Pattar, Veeranan Yadav P., Neha Jain, Ishaan Saha, Aaquib Nazir, Preeti Dhir, Miral Mehta, Jugajyoti Pathi

**Affiliations:** 1 Department of Oral and Maxillofacial Surgery & Implantology, The Oxford dental College, Bengaluru, India; 2 Department of Orthopaedics, Government Royapettah Hospital, Chennai, Tamil Nadu, India; 3 Department of Oral and Maxillofacial Surgery, People's Dental Academy, Bhopal, Madhya Pradesh, India; 4 Department of Dental Surgery, Sri Ramachandra Dental College & Hospital, SRIHER, Porur, Chennai, Tamil Nadu, India; 5 Department of Dentistry, SNM District Hospital, Leh, UT Ladakh, India; 6 Department of Oral Medicine and Radiology, Dasmesh Institute of Research and Dental Sciences, Faridkot, Punjab, India; 7 Department of Pediatric and Preventive Dentistry, Karnavati School of Dentistry, Karnavati University, Gandhinagar, Gujarat, India; 8 Department of Oral & Maxillofacial Surgery, Kalinga Institute of Dental Sciences, KIIT Deemed to be University, Bhubaneswar, Odisha, India

**Keywords:** Fracture healing, osteoporosis, diabetes mellitus, mechanical loading, bone regeneration

## Abstract

The independent and combined effects of osteoporosis and diabetes on mandibular and tibial fracture healing under varied mechanical loading in a rat model (n=120) is of interest. Osteoporosis and diabetes each impaired healing, with comorbid rats showing the most severe reductions in BMD (Bone Mineral Density) and biomechanical strength. Cyclic loading worsened outcomes in diabetic rats, while reduced loading benefited osteoporotic fractures. Disarranged angiogenesis and collagen organization in comorbid groups is confirmed histologically. Thus, we show the need to customize various procedures in patients with systemic comorbidities.

## Background:

The process of healing bone fractures is complex and involves several stages. It can be influenced by many local and systemic health conditions [1]. In the disease of osteoporosis, there negligible bone mass, in other words, there has been qualitative damage to the microarchitecture and quality of bone tissue, which results in delayed and compromised fracture repair due to reduced osteoblast activity leading to late mineralization of callus [2]. Diabetes mellitus delays healing through several mechanisms, including glycaemic-induced increased oxidative stress, increased advanced glycation end-products (AGEs) and reduced angiogenesis [3]. Both diseases have a high prevalence in the world population: osteoporosis with 200 million people among all populations and diabetes with 537 million adults [4, 5]. Mechanical loading is a major positive regulator of bone healing that promotes mesenchymal stem differentiation and matrix deposition via mechanotransduction pathways [6]. Optimal loading enhances callus formation and overly large loading or insufficient loading lead to non-union or delayed healing [7]. Recent findings indicated that osteoporosis reduced sensitivity in bones regarding mechanical stimuli, while diabetes modified extracellular matrix integrity as well as further impairing load-adaptive responses [8, 9]. However, an interaction between these comorbid conditions and mechanical loading has yet to be adequately assessed and compared on facial versus long bones which differ by embryonic origin, vascularity and biomechanical demand [10]. Also, it is very important to know the differential healing responses of facial and long bones because they are different anatomically and functionally. The mandible forms from neural crest cells by intramembranous ossification and the tibia forms by endochondral ossification [5]. This makes them different in healing capacity, vascularization patterns and loading environments. For example, mandibular fractures are exposed to complicated masticatory forces while tibial fractures are exposed to axial and torsional loads induced by weight bearing. This biomechanical diversity may modulate differently the healing response under a systemic condition such as osteoporosis and diabetes but is not yet well addressed in studies [6]. Furthermore, the effect of mechanical loading associated with systemic diseases on bone healing is an expanding field of interest. Mechanical loading has been shown to drive osteogenesis through mechanotransduction pathways involving integrin activation, Wnt/β-catenin signaling and upregulation of osteogenic transcription factors (RUNX2, Osterix) [7]. But osteoporosis attenuates these responses due to loss of mechanosensitivity by osteocytes and shift in bone remodelling rates. In diabetes, hyperglycaemia impairs mechanosensitive signalling pathways, thereby blunting the anabolic response to load. These disruptions suggest that mechanical loading, which is typically beneficial in fracture healing, could have variable or even detrimental effects in patients with comorbid osteoporosis and diabetes [8]. More knowledge on the positive interactive impacts of systemic comorbidities and mechanics on bone regeneration will help develop more effective treatment approaches and rehabilitation strategies to enhance clinical fracture management [6]. Research gaps in the field are the absence of: (1) systemic data on the comparative healing of facial vs. long bones in the comorbid states, (2) knowledge of how loading regimens modulate healing in osteoporosis-diabetes and (3) mechanistic under- standing of the impaired mechanotransduction in these conditions [9, 10-11]. Therefore, it is of interest to assess the independent and interactions effects of osteoporosis and diabetes on rat mandibular and tibial fracture healing under static, dynamic and suboptimal loading conditions and to establish the basis for a future clinical targeting.

## Materials and Methods:

## Study design:

A 12-week randomized controlled animal trial using 120 female Sprague-Dawley rats (12 weeks old, 250±20 g). Groups (n=30/group) included: (1) Control (sham-operated), (2) Osteoporosis (ovariectomized, OVX), (3) Diabetes (streptozotocin-induced, STZ) and (4) Comorbid (OVX+STZ). Osteoporosis was induced via bilateral ovariectomy at Week 0, confirmed by dual-energy X-ray absorptiometry (DXA) showing >20% BMD reduction. Diabetes was induced at Week 4 via intraperitoneal STZ (55 mg/kg), with blood glucose >250 mg/dL confirming hyperglycemia.

## Fracture model and loading:

Anesthesia was administered and standardized mid-diaphyseal fractures were induced in the right tibial and mandibular regions at 8 weeks. External fixators were attached and rats were divided into loading groups (n=10/group): (a) Static (no load), (b) Cyclic (2N, 2Hz, 15 min/day) and (c) Reduced (50% weight loading). Loading started at 8 weeks following the operation.

## Outcome measures:

[1] Micro-CT: Callus volume (mm^3^), BMD (mgHA/cm^3^) and trabecular thickness (Tb.Th, mm) at endpoint.

[2] Biomechanics: Three-point bending (tibia) and compression (mandible) tests for failure load (N) and stiffness (N/mm).

[3] Histology: H & E and Masson's trichrome staining for cartilage/bone ratio, fibrous tissue (%) and angiogenesis (vessels/mm^2^).

## Statistical analysis:

Data expressed as mean ± SD. Two-way ANOVA compared group and loading effects, with Tukey's post-hoc test. Significance: p<0.05. Power analysis (α=0.05, β=0.8) determined sample size.

## Results:

Comorbid rats exhibited significant weight loss (18.3±3.2% vs. control, p<0.001) and hyperglycaemia (382±45 mg/dL). Mortality was 5% (n=6), primarily in diabetic groups. Osteoporosis reduced callus volume (32.4±4.1% vs. control, p<0.001) and BMD (28.7±3.5%, p<0.001). Diabetes decreased BMD (24.1±3.8%, p<0.001) and Tb.Th (21.6±2.9%, p<0.01). Comorbidity amplified deficits (BMD: 45.6±5.2% reduction, p<0.001). Cyclic loading worsened BMD in diabetic groups (p<0.05), while reduced loading improved osteoporotic callus volume (p<0.05) (Table 1 - see PDF). Similar trends but less severe; comorbidity reduced BMD by 31.2±4.3% (p<0.001). Tibial failure load was lowest in comorbid groups (52.7±6.8 N vs. control 98.4±8.2 N, p<0.001). Mandibular stiffness decreased by 39.8±4.5% in comorbidity (p<0.001). Cyclic loading reduced failure load in diabetes (p<0.05). Comorbid fractures showed 41.3±5.1% fibrous tissue (vs. 12.4±2.1% control, p<0.001), reduced angiogenesis (8.2±1.5 vs. 18.7±2.3 vessels/mm^2^, p<0.001) and disorganized collagen. Reduced loading enhanced mineralization in osteoporosis (p<0.05).

## Discussion:

This study demonstrates that osteoporosis and diabetes independently and synergistically impair fracture healing in both facial and long bones, with mechanical loading critically modulating outcomes. The 45.6% reduction in tibial BMD and 39.8% decrease in mandibular failure load in comorbid rats underscore the severe impact of combined pathology, aligning with clinical observations of delayed unions in osteoporotic-diabetic patients [12, 13]. Osteoporosis primarily disrupted early callus formation, reducing volume and BMD by 32.4% and 28.7%, respectively. This corroborates prior findings that oestrogen deficiency suppresses osteoblast proliferation and Wnt/β-catenin signalling, diminishing mechanosensitivity [14]. Notably, reduced loading improved outcomes in osteoporotic groups, suggest that low-magnitude mechanical stimuli may enhance anabolic responses in compromised bone, as seen in studies of low-intensity vibration therapy [15]. Diabetes induced significant fibrosis (41.3% increase) and impaired angiogenesis, consistent with hyperglycaemia-driven AGE accumulation and VEGF suppression [16]. Cyclic loading exacerbated these deficits, potentially due to repetitive microdamage overwhelming repair capacity in a metabolically stressed environment [17]. This contrasts with healthy bone, where cyclic loading promotes healing via fluid shear stress and nitric oxide signalling [18]. The comorbid group exhibited the most severe deficits, with synergistic reductions in BMD and biomechanical strength. Histology revealed disorganized collagen and poor vascularization, reflecting interactions between osteoporosis-driven bone fragility and diabetes-induced microangiopathy [19]. Mandibular fractures showed less impairment than tibiae, likely due to higher vascularity and distinct embryonic origins (neural crest vs. mesoderm), supporting site-specific healing responses [20]. Limitations include the use of an animal model, which may not fully replicate human comorbidities. Future studies should explore molecular mechanisms (e.g., RAGE/NF-κB signalling) and validate findings in clinical cohorts.

## Conclusion:

Osteoporosis and diabetes independently impair fracture healing in facial and long bones, with comorbidity causing synergistic deterioration in bone quality and mechanical strength. Loading conditions critically modulate outcomes: cyclic loading exacerbates delays in diabetes, while reduced loading benefits osteoporosis. Thus, we show the need for comorbidity-specific rehabilitation protocols and mechanoadaptive therapies to optimize fracture repair.
